# A novel molecule targeting neutrophil-mediated B-1a cell trogocytosis attenuates sepsis-induced acute lung injury

**DOI:** 10.3389/fimmu.2025.1597887

**Published:** 2025-06-11

**Authors:** Yuichi Akama, Jespar Chen, Alok Jha, Yongchan Lee, Gaifeng Ma, Jingsong Li, Atsushi Murao, Ping Wang, Monowar Aziz

**Affiliations:** ^1^ Center for Immunology and Inflammation, The Feinstein Institutes for Medical Research, Manhasset, NY, United States; ^2^ Departments of Surgery and Molecular Medicine, Zucker School of Medicine at Hofstra/Northwell, Manhasset, NY, United States

**Keywords:** B-1a cell, neutrophil, sepsis, Siglec-G, CD47, trogocytosis, peptide

## Abstract

Sepsis is a dysregulated immune response to infection. B-1a cells play a crucial role in maintaining immuno-physiologic homeostasis. Sialic acid-binding immunoglobulin-like lectin G (Siglec-G) regulates B-1a cell’s behavior and function. Trogocytosis is the process by which one cell acquires portions of another cell’s plasma membrane and cytoplasm through direct contact. During sepsis, neutrophils accumulate in the lungs and serosal cavities, while B-1a cells decrease. We hypothesized that neutrophil-mediated trogocytosis causes B-1a cell depletion in sepsis, and that targeting this process could preserve B-1a cells and attenuate sepsis-induced acute lung injury (ALI). Sepsis was induced in mice by cecal ligation and puncture (CLP). Twenty hours after CLP, B-1a cells (CD19^+^B220^lo/-^CD23^-^CD5^+^) in the pleural and peritoneal cavities were quantified, and neutrophil engulfment of B-1a cells as well as trogocytosis were assessed. We also examine the interaction between Siglec-G and the “don’t-eat-me” signal receptor, CD47. Our data showed that B-1a cell numbers and frequencies in the pleural and peritoneal cavities were significantly decreased in sepsis. Neutrophils co-cultured with B-1a cells significantly increased B-1a cell internalization via trogocytosis. We observed a strong binding interaction between Siglec-G and CD47, which facilitates neutrophil-mediated trogocytosis by compromising CD47 function. We discovered a novel 11-aa therapeutic peptide, named Compound 11 (C11), derived from the CD47 region interacting with Siglec-G. C11 effectively preserved B-1a cell populations, significantly reduced pro-inflammatory cytokine levels, alleviated ALI, and improved survival in sepsis. Our findings highlight the Siglec-G/CD47 axis on B-1a cells as a key regulator of neutrophil-mediated B-1a cell depletion. Targeting this pathway with C11 represents a promising therapeutic strategy to mitigate immune dysregulation and improve sepsis outcomes.

## Introduction

Sepsis is a life-threatening condition caused by organ dysfunction due to a dysregulated immune response to infection (1). A hallmark of sepsis is the early excessive production of pro-inflammatory cytokines, leading to systemic inflammation and organ injury (2). In clinical settings, elevated early pro-inflammatory responses correlate with disease severity (3). Consequently, effective suppression of excessive inflammation remains a key target for sepsis treatment. Despite extensive research, therapeutic strategies aimed at antagonizing individual pro-inflammatory cytokines have not significantly improved patient survival (4). The complexity of the inflammatory cytokine network presents a major challenge in developing effective treatments (4). Current sepsis treatments focus on infection control and supportive care, yet no FDA-approved therapies exist that specifically address the underlying mechanisms driving sepsis and its complications, including acute lung injury (ALI).

B-1a cells are a unique subpopulation of B cells with profound immunoregulatory properties (5). These cells are primarily present in the serosal cavities, like pleural and peritoneal cavities. They produce large amounts of the anti-inflammatory cytokine IL-10 either spontaneously or in response to infection, contributing to immune homeostasis and resolution of inflammation (6). Additionally, B-1a cells exhibit innate-like functions, playing a critical role in the early defense against invading pathogens by secreting natural antibodies (IgM), which aid in host protection against acute infections and help lower bacterial burden (7). Recent studies have demonstrated the beneficial role of B-1a cells in sepsis, as they help regulate systemic levels of pro-inflammatory cytokines and bacterial loads, thereby mitigating organ injury, including ALI (6, 8, 9). Importantly, adoptive transfer of B-1a cells in multiple sepsis models has been shown to improve survival rates (6, 9). These findings highlight the pivotal role of B-1a cells in maintaining immune homeostasis during sepsis and suggest that they may serve as a potential therapeutic target for modulating immune responses and reducing sepsis-induced organ injury.

Sialic acid-binding immunoglobulin-like lectin (Siglec)-G is a member of the CD33-related Siglec family and is highly expressed on B-1a cells (10). It plays a critical role in regulating B-1a cell’s behavior and functions (5, 10, 11). Notably, Siglec-G can bind sialic acid-modified substrates through immunoglobulin-like receptors and interact with endogenous ligands such as B cell receptor (BCR) (10) and CD24 (12), thereby exerting immunoregulatory effects on B cells. Siglec-G interacts with BCR to regulate exaggerated proliferation of B-1a cells (10). Moreover, Siglec-G controls B-1a cell migration by interacting with CXCR4 to maintain B-1a cell pool in the serosal cavity during inflammation (11). Despite its essential role in immune modulation, proliferation, and migration, the full extent of Siglec-G’s interactions with endogenous molecules to maintain B-1a cell homeostasis in sepsis remains poorly understood.

CD47, a member of the immunoglobulin superfamily, is known as a “don’t-eat-me” signal for phagocytes (13). It is broadly expressed on lymphocytes and other cell types, where it negatively regulates phagocyte activity by engaging signal regulatory protein α (SIRPα), a receptor expressed on phagocytes such as neutrophils and macrophages (13–16). Indeed, anti-CD47 treatment has been shown to deplete CD19^+^ B cells under steady-state conditions (17), indicating that CD47 plays a crucial role in B cell maintenance by protecting them from uncontrolled phagocytosis. Additionally, CD47 interacts not only with SIRPα but also with other molecules on the same cell surface, such as integrin family members, further regulating immune cell signaling (16). Interestingly, CD47 is a glycoprotein that may carry sialylated glycans (18, 19), suggesting a potential interaction with Siglec-G.

Trogocytosis is a biological process in which a cell nibbles membrane fragments from another cell. The SIRPα-CD47 signaling pathway plays a critical role in regulating trogocytosis, preventing excessive or uncontrolled cell nibbling (14, 15). Notably, a recent study demonstrated that neutrophil-mediated trogocytosis can induce cell death, leading to the loss of cytoplasmic material (20). Despite the well-established role of neutrophils as the most abundant immune cells in sepsis, there are no studies investigating neutrophil-mediated trogocytosis of B-1a cells in this context.

In this study, we hypothesize that Siglec-G/CD47 interactions on B-1a cells enhance neutrophil-mediated B-1a cell trogocytosis, thereby contributing to B-1a cell depletion in sepsis. Understanding this mechanism will provide new insights into B-1a cell loss and its broader implications for immune homeostasis and dysregulation in sepsis.

## Materials and methods

### Mice

Male 8–12-week-old wild-type (WT) C57BL/6 mice were purchased from Charles River (Charles River, Wilmington, MA). All mice were maintained under specific pathogen-free conditions in a temperature-controlled room with a 12 h light/dark cycle and provided with standard laboratory chow and water. All experiments were performed in accordance with the guidelines for the use of experimental animals by the National Institutes of Health and were approved by the Institutional Animal Care and Use Committee (IACUC) of the Feinstein Institutes for Medical Research.

### *In silico* prediction of protein-protein interactions and peptide synthesis

The amino acid sequences of mouse CD47 (Q61735) and SiglecG (Q80ZE3) were retrieved from the Uniprot database. The structure models were generated by using Iterative Threading ASSembly Refinement approach of ITASSER tool (21) that is based on identifying templates by threading approach to maximize the percentage identity, sequence coverage and confidence. The structure models were refined based on repetitive relaxations by short molecular dynamics simulations for mild (0.6 ps) and aggressive (0.8 ps) relaxations with 4 fs time step after structure perturbations. The structure refinement enhanced certain parameters including Rama favored residues and decrease in poor rotamers. The CD47-SiglecG complex was formed using the protein-protein docking approach. The GRAMMX (22) and ATTRACT (23) tools were used for the docking of CD47 and SiglecG. The docking approach allows simultaneous adjustment of side chain conformations and energy minimization in translational and rotational degrees of freedom of one protein with respect to another protein. In a different approach the potential energy on a grid was calculated for the docking process and then the interactions are calculated by interpolation from nearest grid points. Moreover, the docking process includes several energy minimization steps. The protein-protein interaction between CD47 and SiglecG was calculated using PDBePISA tool (24). The surface area of the interaction interface and thermodynamic parameters were calculated. The complex structure was visualized using PyMOL and Chimera. The CD47-SiglecG complex was analyzed for the interaction interface residues and peptide was designed based on the amino acids involved in interaction at the interaction interface of CD47-SiglecG. The therapeutic peptide, designated as Compound 11 (C11), comprises an 11-amino-acid sequence (126-ELKNRTVSWFS-136) derived from the CD47 sequence and was synthesized by GenScript (Piscataway, NJ). C11 was computationally docked onto Siglec-G using the pepATTRACT tool (25). The interaction between C11 and Siglec-G was measured using PDBePISA tool (24).

### Murine model of polymicrobial sepsis

Mice were anesthetized with 2% isoflurane in oxygen, and sepsis was induced via cecal ligation and puncture (CLP) (26). A 1-cm midline abdominal incision was made to expose the cecum, which was ligated 1-cm proximal to its end using a 4-0 silk suture. The cecum then was perforated with a single through-and-through puncture using a 22-gauge needle, positioned midway between the ligation and the tip of the cecum. A small amount of fecal material was gently extruded from the perforation sites to confirm the patency of the punctures, and the cecum was subsequently returned to the peritoneal cavity. Following abdominal closure, resuscitation was provided via subcutaneous (*s.c.*) injection of 500 µL normal saline. Postoperative analgesia was administered with a single dose of 0.1 mg/kg buprenorphine. To evaluate the effects of the therapeutic peptide (named Compound 11 or C11) (synthesized by GenScript USA Inc., Piscataway, NJ) on sepsis, C57BL/6 mice were assigned to three experimental groups: the Sham group, the CLP group treated with vehicle, and the CLP group treated with C11 (10 mg/kg). C11 was dissolved in DMSO (10 mg/mL) and diluted with saline to a total volume of 500 µL. The peptide was administered once, immediately after surgery, via intraperitoneal (*i.p.*) injection. Mice in the vehicle group received an equivalent volume of DMSO and normal saline via *i.p.* injection. After 20 h, lungs were harvested, and blood was collected via cardiac puncture. For the survival study, animals received a single subcutaneous (*s.c.*) injection of 0.5 mg/kg imipenem at the end of the CLP.

### Isolation of murine B-1a cells from pleural and peritoneal cavities

Mice were euthanized by CO_2_ asphyxiation. Following skin and abdominal incision, the pleural cavity was exposed using a surgical vessel clamp on the xiphoid process (27). 1 mL of PBS with 2% FBS was injected into the pleural cavity through the diaphragm. Using tubing connected to a syringe inserted through the same entry point, the cavity was washed with PBS at least three times, and the fluid collected. After centrifugation (300 × g, 10 min, 4°C), the cell pellet was resuspended in PBS containing 0.5% BSA and 2 mM EDTA. 1 mL of red blood cell (RBC) lysis buffer was added, mixed, and incubated for 2 min at room temperature. 5 mL of RPMI was then added, followed by centrifugation (300 × g, 10 min, 4°C). The cell pellet was resuspended in PBS containing 0.5% BSA and 2 mM EDTA. For isolating peritoneal B-1a cells (9), the peritoneal cavity was lavaged with PBS containing 2% FBS. After centrifugation (300 × g, 10 min, 4°C), the cell pellet was resuspended in PBS containing 0.5% BSA and 2 mM EDTA. B-1a cells were then purified using negative and positive immunomagnetic selection with the B-1a Cell Isolation Kit (Cat. No. 130-097-413, Miltenyi Biotec, Bergisch Gladbach, Germany).

### Isolation of neutrophils

Mice were euthanized using CO_2_ asphyxiation, and neutrophils were isolated from either the bone marrow or the peritoneal cavity of sham or septic mice (9). For bone marrow neutrophil isolation, bone marrow cells were flushed from the femurs and tibias using ice-cold PBS. The collected cells were passed through a sterile 70 μm nylon filter to remove debris and then resuspended in PBS supplemented with 2% fetal bovine serum (FBS). For peritoneal neutrophil isolation, peritoneal lavage cells were collected as described above. Neutrophils were then purified using immunomagnetic negative selection with the EasySep Mouse Neutrophil Enrichment Kit (Cat. No. 19762, STEMCELL Technologies, Vancouver, BC, Canada).

### Detection of B-1a cell and neutrophil numbers

B-1a cell and neutrophil populations were analyzed by flow cytometry (9, 11, 28). Cell suspensions from mice were incubated for 30 mins at 4°C with combinations of monoclonal fluorescently conjugated Abs: CD45-PerCP/Cy5.5 (I3/2.3, Cat. No. 147706, BioLegend, San Diego, CA, USA), B220-APC (RA3-6B2, Cat. No. 103212, BioLegend), CD19-APC/Fire 750 (6D5, Cat. No. 115558, BioLegend), CD5-PE (53-7.3, Cat. No. 100608, BioLegend), CD23-PE/Cy7 (B3B4, Cat. No. 101614, BioLegend), Siglec-G-BV421 (SH1, Cat. No. 163309, BioLegend), CD47-BV421 (miap301, Cat. No. 127527, BioLegend), or Ly6G-PE (1A8, Cat. No. 127608, BioLegend). After that, B-1a cells (CD45^+^CD19^+^B220^lo/-^CD5^+^CD23^-^) (6, 28), and neutrophils (CD45^+^Ly6G^+^) were detected by flow cytometry. Fc block (Cat. No. 156604, BioLegend) was used to prevent nonspecific antibody binding and the cell viability was determined using a Zombie Aqua Fixable Viability Kit (BioLegend). Flow cytometric analysis was performed on a FACSymphony (BD Biosciences, Franklin Lakes, NJ) and data were processed using FlowJo software (BD Biosciences). The absolute number of cells was calculated by using Precision Count Beads (BioLegend) by comparing the number of detected cells to the number of detected beads, following the manufacturer’s instructions.

### Analyzing trogocytosis by confocal imaging and flow cytometry

B-1a cells (target cells) were isolated, and dead cells were removed using the EasySep Dead Cell Removal Kit (Cat. No. 17899, STEMCELL Technologies). The viable B-1a cells were then labeled using the CellTrace CFSE Cell Proliferation Kit (Thermo Fisher Scientific, Waltham, MA) according to the manufacturer’s instructions. Neutrophils (effector cells) were pretreated with an Fc block and stained with Ly6G. The labeled target cells and effector cells were then co-cultured at a target and effector cell ratio of 4:1 in a 96-well plate for 2 h at 37°C and 5% CO_2_. Trogocytosis was evaluated using confocal imaging (LSM900; Zeiss, Oberkochen, Germany) to visualize the interaction between neutrophils and B-1a cells. NucBlue Live ReadyProbes Reagent (Thermo Fisher Scientific) was used for nuclear staining. For quantitative analysis, trogocytosis was assessed by flow cytometry, where CFSE (Carboxyfluorescein succinimidyl ester)-positive events within Ly6G-positive neutrophil populations were quantified as indicators of trogocytosis. B-1a cells and neutrophils were isolated from the peritoneal cavity or bone marrow 4 h after surgery for analysis. To evaluate the effect of C11, the peptide was administered once (10 mg/kg) via *i.p.* injection immediately after CLP operation.

### Live cell imaging

B-1a target cells were labeled with DiO (5 μM; Biotium Inc., Fremont, CA) to stain the cell membrane, while Calcein Red-AM (4 μM; BioLegend) was used to label the cytoplasm of live cells. Labeled target cells were co-incubated with neutrophil effector cells at an effector to target ratio of 4:1 in μ-Slide 8 Well ibiTreat (ibidi GmbH, Gräfelfing, Germany) at 37°C and 5% CO_2_. The cells were maintained in RPMI 1640 (Thermo Fisher Scientific), supplemented with 10% FBS and Penicillin-Streptomycin (100 IU/mL; Thermo Fisher Scientific). Live imaging was initiated within 5 mins after the start of co-incubation and conducted at 2-min intervals using a LSM900 confocal microscope (Zeiss).

### Surface plasmon resonance assay

To examine the direct interaction between recombinant mouse (rm)Siglec-G (Cat. No. 10103-SL-050, R&D Systems, Minneapolis, MN) and rmCD47 (Cat. No. AB231160, Abcam, Cambridge, UK), surface plasmon resonance (SPR), using OpenSPR (Nicoya, Ontario, Canada), was performed. HS-Carboxyl sensor was used; rmCD47 was immobilized on the sensor surface; rmSiglec-G was in the reaction buffer, working as an analyte. Binding reactions were performed in PBS 0.05% P20, pH7.4. The carboxyl sensor was first cleaned by injecting 150 µL of 10 mM HCl, followed by injection of 150 µL of the mixture of 1 aliquot of N-ethyl-N′-[3-diethylaminopropyl]-carbodiimide (EDC) and 1 aliquot of N-hydroxysuccinimide (NHS) to activate the sensor surface. An aliquot of 200 μL of 50 µg/mL of CD47 diluted in 10 mM sodium acetate (pH 5) was injected into flow cell-channel-2 of the sensor for immobilization. Next, 150 µL of 1 M ethylenediamine (pH 8.5) was injected to deactivate the remaining active sites on channel 1and 2. The flow cell-1 was used as a control to evaluate nonspecific binding. The binding analyses were performed at a flow rate of 40 μL per minute at 20°C. To evaluate the binding, the analyte ranging from 100 nM, 250 nM and 500 nM were injected into flow cell-1 and flow cell-2, and the real-time interaction data were analyzed by TraceDrawer (Nicoya). The signals from the control channel (flow cell-1) were subtracted from the channel coated with the ligand (flow cell-2) for all samples. Data were globally fitted for 1:1 binding. For peptide C11 efficacy on Siglec-G binding to CD47, with the same setting as Siglec-G binding with CD47 on HS-carboxyl sensor, peptide C11 at 1 µM preincubated with concentrations of rmSiglec-G 100 nM, 250 nM & 500 nM for 30 minutes and then injected into flow cell-1 and flow cell-2, and real-time interaction were analyzed ab TraceDrawer (Nicoya). The signals from the control channel (flow cell-1) were subtracted from the channel coated with the ligand (flow cell-2) for all samples.

### Colorimetric enzymatic assays and enzyme-linked immunosorbent assay

Blood was collected by heart puncture. Plasma levels of organ injury markers (ALT: alanine transaminase, AST: aspartate transferase, and LDH: lactate dehydrogenase) were assessed by using colorimetric enzymatic assays (MedTest Dx, Canton, MI) according to the manufacturer’s protocol. Plasma levels of pro-inflammatory cytokines (TNFα: tumor necrosis factor α, and IL-6: interleukin-6) were measured by using mouse-specific ELISA kits (BD Biosciences).

### RNA isolation and real-time quantitative PCR

Total RNA was extracted from lungs using a TRIzol reagent (Thermo Fisher Scientific) according to the manufacturer’s instructions. cDNA was synthesized using M-MLV reverse transcriptase (Thermo Fisher Scientific). Quantitative real-time PCR (qPCR) was performed using SYBR Green PCR Master Mix (Thermo Fisher Scientific) with a StepOnePlus Real-Time PCR thermocycler (Thermo Fisher Scientific). Mouse β-actin served as an endogenous control to normalize mRNA levels using the comparative Ct method. The primer sequences used in this study, along with their corresponding gene targets, are listed in **Supplementary Table 1**.

### Lung histopathology

Lung tissues were fixed in 10% formalin, embedded in paraffin, sectioned into 5-μm-thick slices, and stained with hematoxylin and eosin (H&E). Histological assessment of lung injury was performed using the scoring system of the American Thoracic Society (29). Scores, ranged from 0 to 1, were assigned based on the following features: neutrophils in the alveolar space, neutrophils in the interstitial space, hyaline membranes, proteinaceous debris filling the airspaces, and alveolar septal thickening. Lung fields at ×400 magnification were scored and averaged for analyses.

### Lung myeloperoxidase

Approximately 50 to 100 mg of septic lung tissue, previously snap-frozen in liquid nitrogen and pulverized into a fine powder, was homogenized in potassium phosphate (KPO_4_) buffer containing 0.5% hexadecyltrimethylammonium bromide (HTAB; MilliporeSigma, Burlington, MA). Homogenization was performed using sonication while keeping the samples on ice to preserve enzymatic activity. To enhance cell lysis and release of MPO, the samples underwent two freeze-thaw cycles before being centrifuged to collect the resulting supernatant. The MPO activity assay was conducted in a 96-well plate by adding the supernatant to a phosphate buffer containing O-dianisidine hydrochloride (MilliporeSigma) and hydrogen peroxide (H_2_O_2_; Thermo Fisher Scientific). The enzymatic reaction was monitored by measuring light absorbance at 460 nm over a five-minute period. MPO activity was expressed as units per gram of tissue, where one unit was defined as the change in absorbance per minute.

### Lung wet and dry ratio

Lung wet and dry weights were measured as previously described (30). Briefly, the left lung was excised and immediately weighed to determine the wet lung weight. The lung samples were then dried at 65°C for 48 h until a constant weight was achieved, after which they were weighed again to determine the dry weight. The wet-to-dry weight ratio was calculated as an indicator of pulmonary edema.

### Terminal deoxynucleotidyl transferase dUTP nick end labeling assay

To determine cellular apoptosis we used a TUNEL assay kit (Roche Diagnostics, Indianapolis, IN) using 5-μm lung sections by following the manufacturer’s instructions. 4’,6’-diamidino-2-phenylindole (DAPI; Vector Laboratories Inc., Newark, CA) was used for nuclear staining. The TUNEL-positive cells were assessed by LSM900 confocal microscope (Zeiss) microscopes. The number of apoptotic cells/fields was quantified using ImageJ, Fiji software (version 1.53k).

### Statistical analysis

All statistical analyses were performed using Prism 10 (GraphPad Software, San Diego, CA). Data are expressed as the mean ± standard error of the mean (SEM), and p-values < 0.05 were considered statistically significant. A two-tailed Student’s t-test was used for comparisons between the two groups. For comparisons among multiple groups, one-way ANOVA followed by Tukey’s *post-hoc* test was applied. All experiments were performed at least twice to ensure reproducibility. For survival analysis, data were assessed using the Kaplan-Meier estimator and compared using the log-rank test.

## Results

### Sepsis decreases B-1a cells

**Figure 1:**
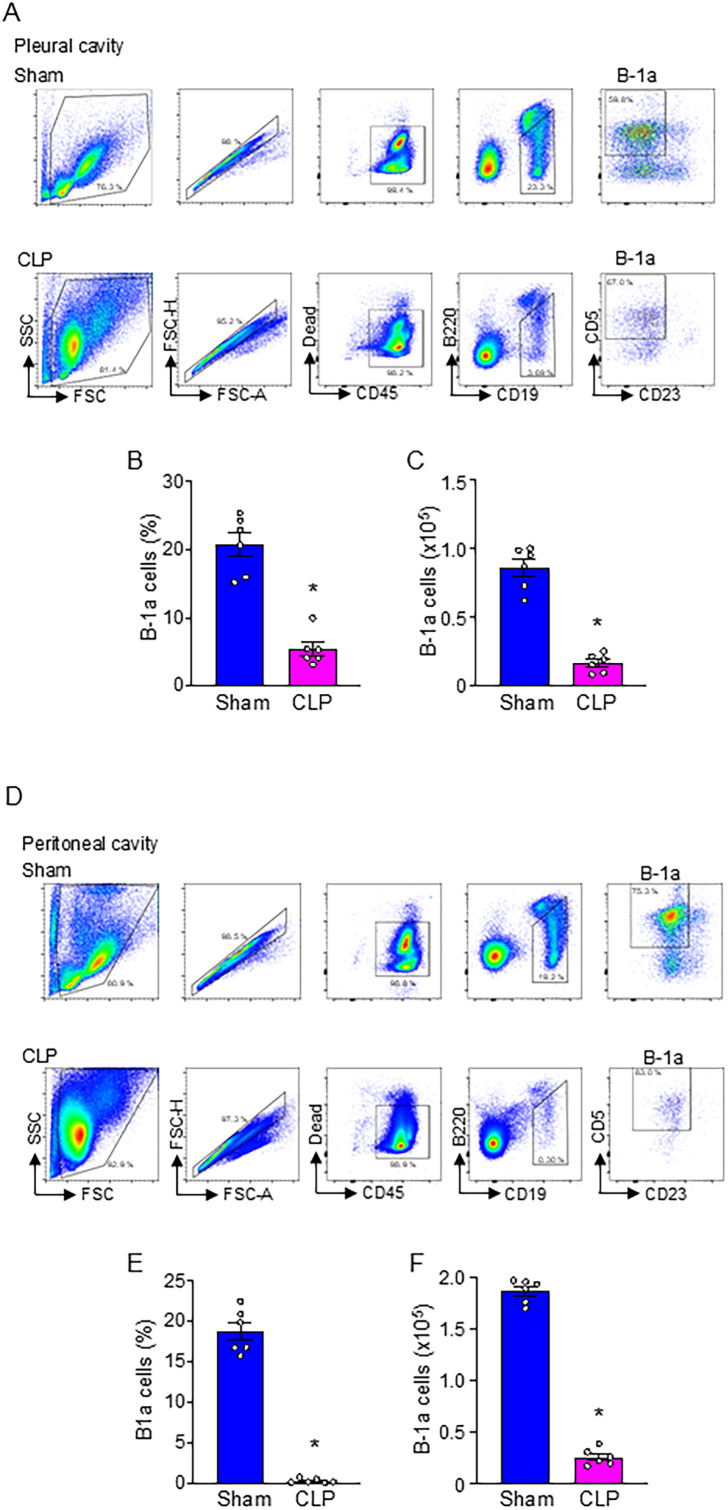
B-1a cells are significantly decreased in sepsis. Sepsis was induced in mice using cecal ligation and puncture (CLP). B-1a cells from **(A-C)** pleural and **(D-F)** peritoneal cavities were collected at 20 h after surgery to determine B-1a cells frequency (B-1a cell within CD45^+^ cells) and numbers. Representative gating strategy of flow cytometry plots for detecting live B-1a cells (CD45^+^CD19^+^B220^lo/-^CD5^+^CD23^-^) from **(A)** pleural and **(D)** peritoneal cavities. **(B, E)** The frequency and **(C, F)** absolute number of B-1a cells in sham and CLP were shown. Data represent the mean ± SEM (n = 6/group). Experiments were performed twice, and all data were analyzed. The groups were compared by Student’s t-test. *p < 0.05 vs. Sham.

We induced sepsis in WT mice by CLP and collected pleural and peritoneal cavity cells at 20 h after surgery to assess B-1a cell frequency and numbers. The results demonstrated a significant reduction in both the frequency and numbers of B-1a cells in the pleural cavities (**Figures 1A-C**) and peritoneal cavities (**Figures 1D-F**). Specifically, the frequency of B-1a cells declined by 74.2% and 97.9% in the pleural (**Figure 1B**) and peritoneal cavities (**Figure 1E**), respectively. Similarly, the absolute number of B-1a cells decreased by 81.2% in the pleural (**Figure 1C**) and 86.1% in the peritoneal cavities (**Figure 1F**). The findings that B-1a cell populations are markedly depleted in sepsis suggest a critical loss of their immunoregulatory function.

### Neutrophils engulf live B-1a cells via trogocytosis in sepsis

**Figure 2:**
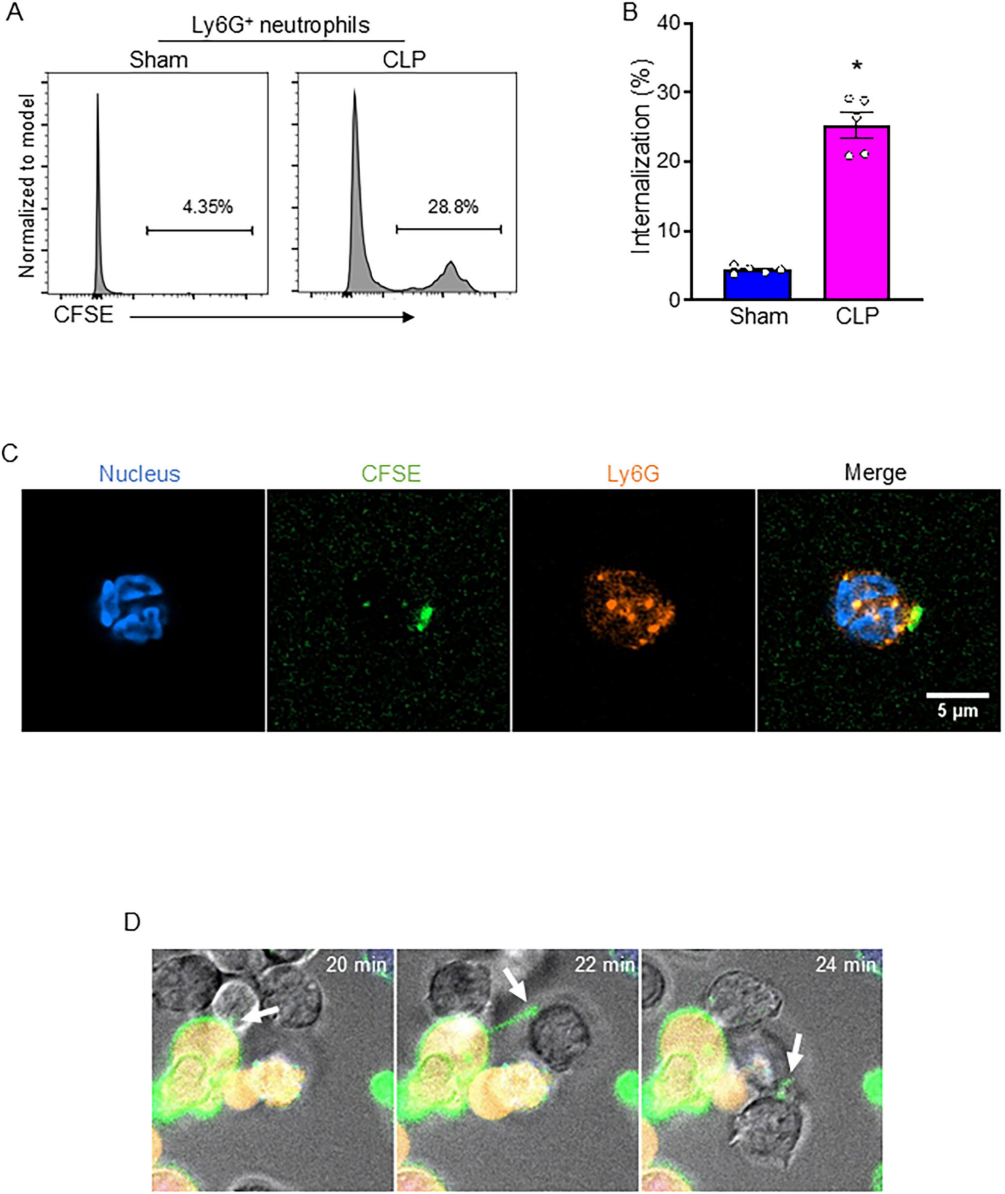
Neutrophil-mediated B-1a cell trogocytosis in sepsis. Sepsis was induced in mice using cecal ligation and puncture (CLP). B-1a cells and neutrophils were collected 4 h after CLP surgery for analysis. Engulfment of B-1a cells/debris was assessed by flow cytometry, where CFSE-positive events within Ly6G-positive neutrophil populations were quantified. **(A)** Representative histogram and **(B)** frequency of sham and CLP were shown. Data represent the mean ± SEM (n = 5/group). Experiments were performed twice, and all data were analyzed. The groups were compared by Student’s t-test. *p < 0.05 vs. Sham. Trogocytosis was evaluated using confocal imaging to visualize the interaction between neutrophils and B-1a cells. **(C)** Immunofluorescence staining showed neutrophils stained with Ly6G (orange) and nuclear stain (blue), while fragments of B-1a cells appear CFSE-positive (green). Scale bar: 5 μm. **(D)** Live-cell imaging was performed to evaluate neutrophil-mediated B-1a cell trogocytosis (arrows), where B-1a cells were labeled with DiO (cell membrane, green), and Calcein Red-AM (cytoplasm of live cells, red), while neutrophils remained unstained.

During sepsis, substantial numbers of neutrophils accumulated in serosal cavities and various organs (11). We first sought to determine whether neutrophils engulf live B-1a cells. To do this, we co-cultured CFSE-labeled B-1a cells (after removing dead cells) with Ly6G-labeled neutrophils and assessed B-1a cell engulfment by quantifying CFSE-positive events within the Ly6G-positive neutrophil population using flow cytometry. We found that neutrophil-mediated internalization of B-1a cells was significantly increased in sepsis (**Figures 2A, B**). To visually confirm this internalization, we performed confocal microscopy. Ly6G staining and nuclear staining confirmed the characteristic nuclear morphology of neutrophils. Interestingly, we observed CFSE-positive fragments at the periphery of neutrophils, with some CFSE signal also present intracellularly, indicating B-1a cell trogocytosis—a piecemeal biting process by neutrophils (**Figure 2C**, **Supplementary Figure 1-2**). This trogocytosis was observed when B-1a cells isolated from septic mice were co-cultured with neutrophils from septic mice. Furthermore, live-cell imaging revealed neutrophils nibbling on the membranes of live B-1a cells (**Figure 2D**), further supporting the occurrence of trogocytosis. These findings provide direct evidence of neutrophil-mediated trogocytosis of live B-1a cells, suggesting that this process is not a physiological mechanism like efferocytosis, which clears dead B-1a cells in sepsis.

### Siglec-G interacts with CD47

**Figure 3:**
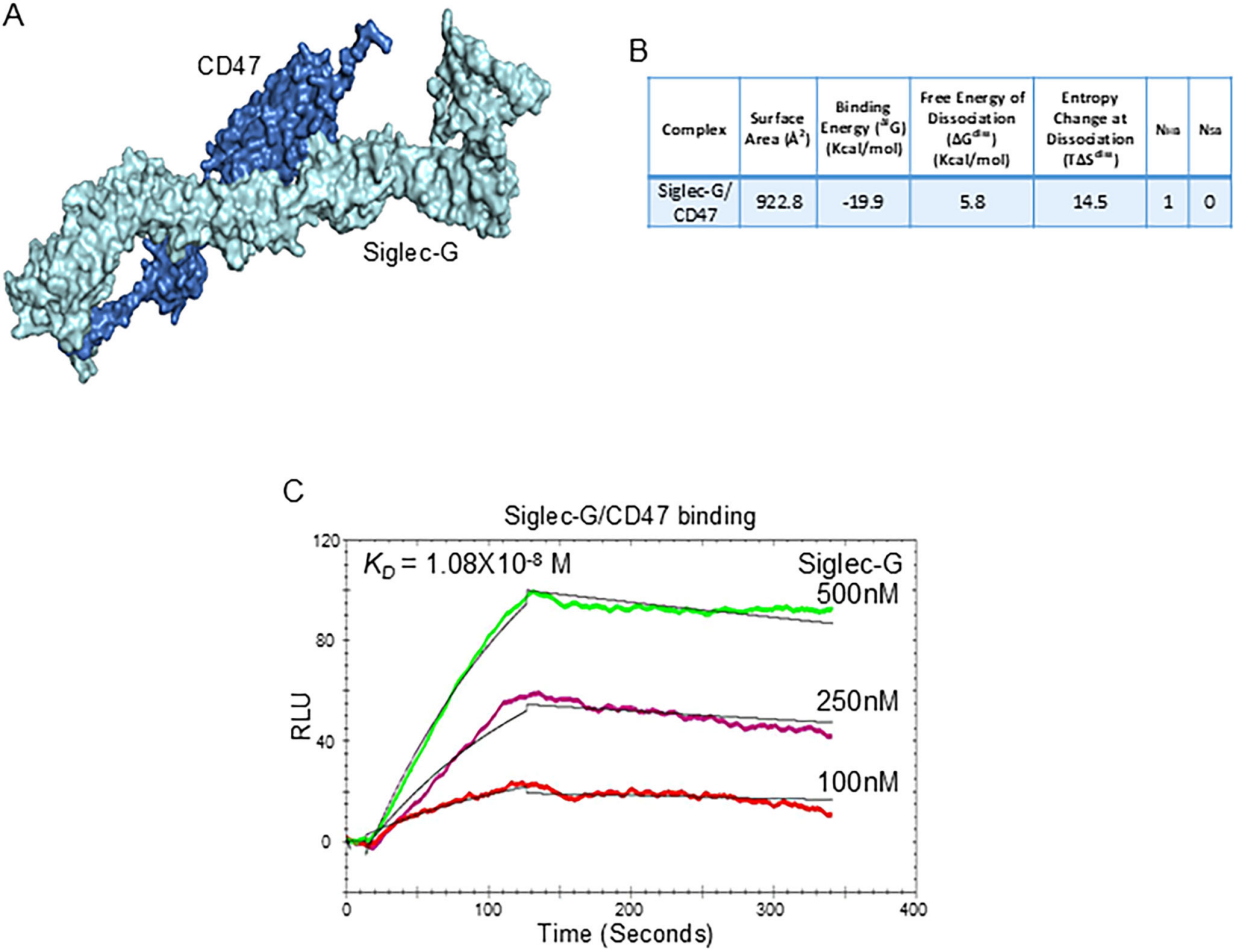
The Siglec-G and CD47 interaction on B-1a cells. **(A)** Computational model illustrating the interaction between Siglec-G (light green) and CD47 (blue). **(B)** Computational prediction of molecular binding between Siglec-G and CD47. **(C)** BIAcore analysis was conducted to assess the binding affinity between recombinant murine Siglec-G and CD47.

We first confirmed that B-1a cells express Siglec-G and CD47 under normal and in sepsis conditions (**Supplementary Figures 3A-F**). Next, we aimed to elucidate whether Siglec-G interacts with CD47. We performed computational modeling to reveal any interaction between Siglec-G and CD47 and identified a strong binding affinity between Siglec-G and CD47 with a predicted binding free energy (^Δi^G) of -19.9 kcal/Mol (**Figures 3A, B**). Subsequently, to further validate this interaction, we performed surface plasmon resonance (SPR) assay, also known as BIAcore assay, using recombinant mouse (rm) Siglec-G and rmCD47. Consistent with the computational analysis, our data revealed a strong interaction between Siglec-G and CD47, with a dissociation constant (*K_D_*) of 1.08 × 10^-8^ M (**Figure 3C**). These findings suggest that Siglec-G strongly binds to CD47, and this binding might interfere with the CD47 “don’t eat me” signal.

### Discovery of C11 and its impact on B-1a cell trogocytosis in sepsis

**Figure 4:**
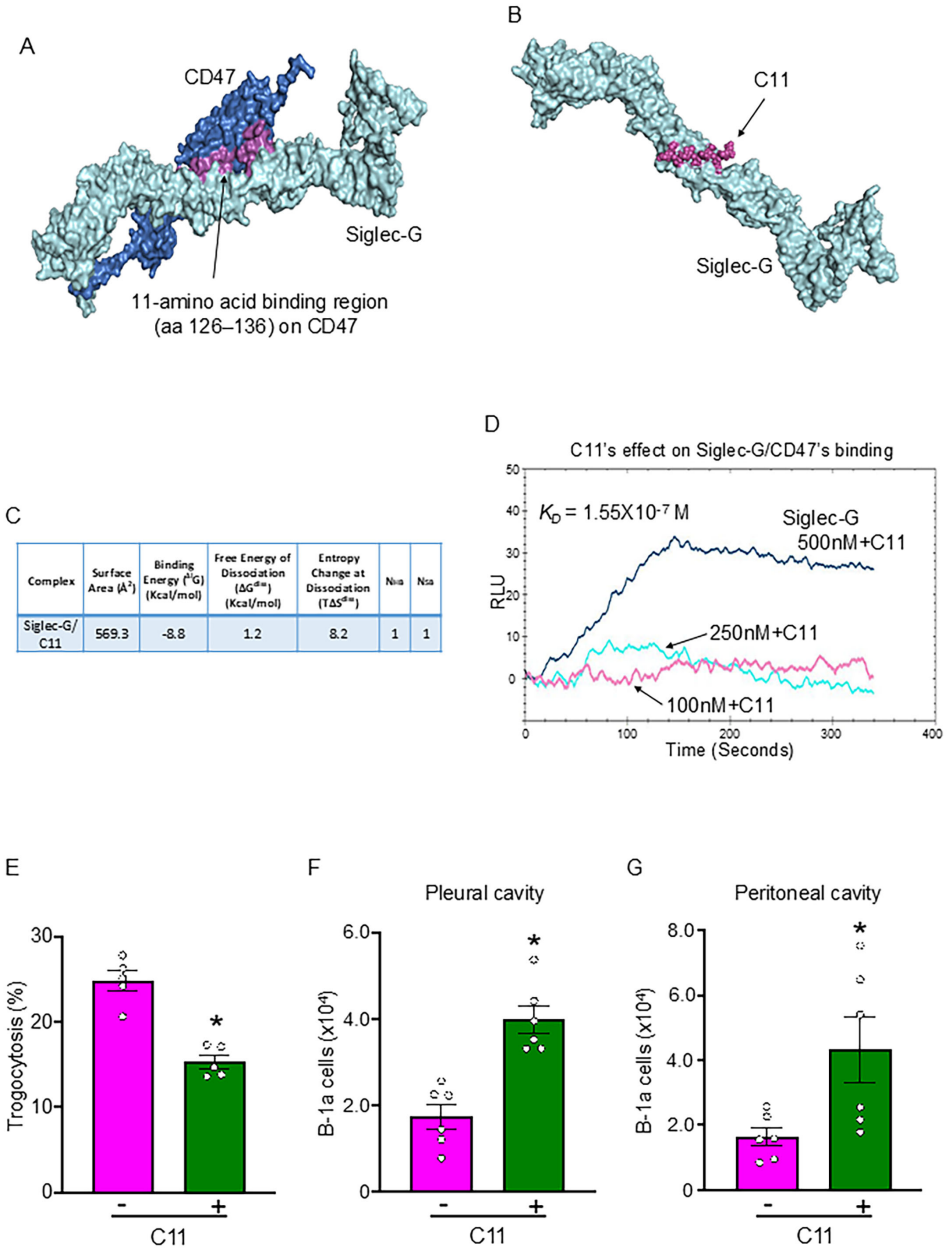
C11 blocks the Siglec-G and CD47 interaction, thereby inhibiting neutrophil-mediated trogocytosis to B-1a cells in sepsis. **(A)** Identification of a putative 11-amino acid binding region (aa 126–136) (purple) on CD47 (blue) for Siglec-G (light green). **(B)** Computational model illustrating the interaction between Siglec-G (green) and C11 (purple). **(C)** Computational prediction of molecular binding between Siglec-G and C11. **(D)** BIAcore analysis demonstrating the efficacy of C11 in inhibiting the Siglec-G/CD47 interaction. **(E)** B-1a cells and neutrophils were isolated 4 h after CLP, and trogocytosis was assessed by flow cytometry. C11 (10 mg/kg) was administered intraperitoneally (*i.p.*) immediately after CLP to evaluate its inhibitory effect on neutrophil-mediated trogocytosis. **(F, G)** The absolute number of B-1a cells in the pleural and peritoneal cavities was quantified 20 h after surgery using flow cytometry to determine the protective effect of C11 on B-1a cell depletion in sepsis. Data represent the mean ± SEM (n = 5-6/group). Experiments were performed twice, and all data were analyzed. The groups were compared by Student’s t-test. *p < 0.05 vs. C11 (-).

Using computational modeling, we identified the region of CD47 with which Siglec-G interacts. A putative 11-amino acid binding region (aa 126–136) on CD47 for Siglec-G was identified (**Figure 4A**). Based on this, we designed Compound 11 (C11; ELKNRTVSWFS), which mimics CD47’s aa 126–136 sequence. Computational modeling revealed a strong binding affinity of C11 for Siglec-G (**Figures 4B, C**). As shown in **Figure 3C**, Siglec-G exhibits a strong binding affinity for CD47 (*K*_D_ = 1.08 × 10^−8^ M). Interestingly, the binding affinity between Siglec-G and CD47 decreased 10-fold (*K*_D_ = 1.55 × 10^−7^ M) in the presence of 1 µM C11 (**Figure 4D**). Thus, by blocking the Siglec-G/CD47 interaction, C11 aims to prevent neutrophil-mediated trogocytosis of B-1a cells, thereby preserving their immunomodulatory function and mitigating sepsis-induced inflammation. As expected, C11 treatment significantly reduced neutrophil-mediated B-1a cell trogocytosis (**Figure 4E**) and effectively preserved B-1a cell numbers in the pleural and peritoneal cavities during sepsis (**Figures 4F, G**). These findings suggest that C11 disrupts Siglec-G/CD47 interactions and protects B-1a cells, demonstrating its potential as a therapeutic strategy for regulating neutrophil-mediated trogocytosis in sepsis.

### C11 attenuates systemic inflammation and ALI, and improves survival in sepsis

**Figure 5:**
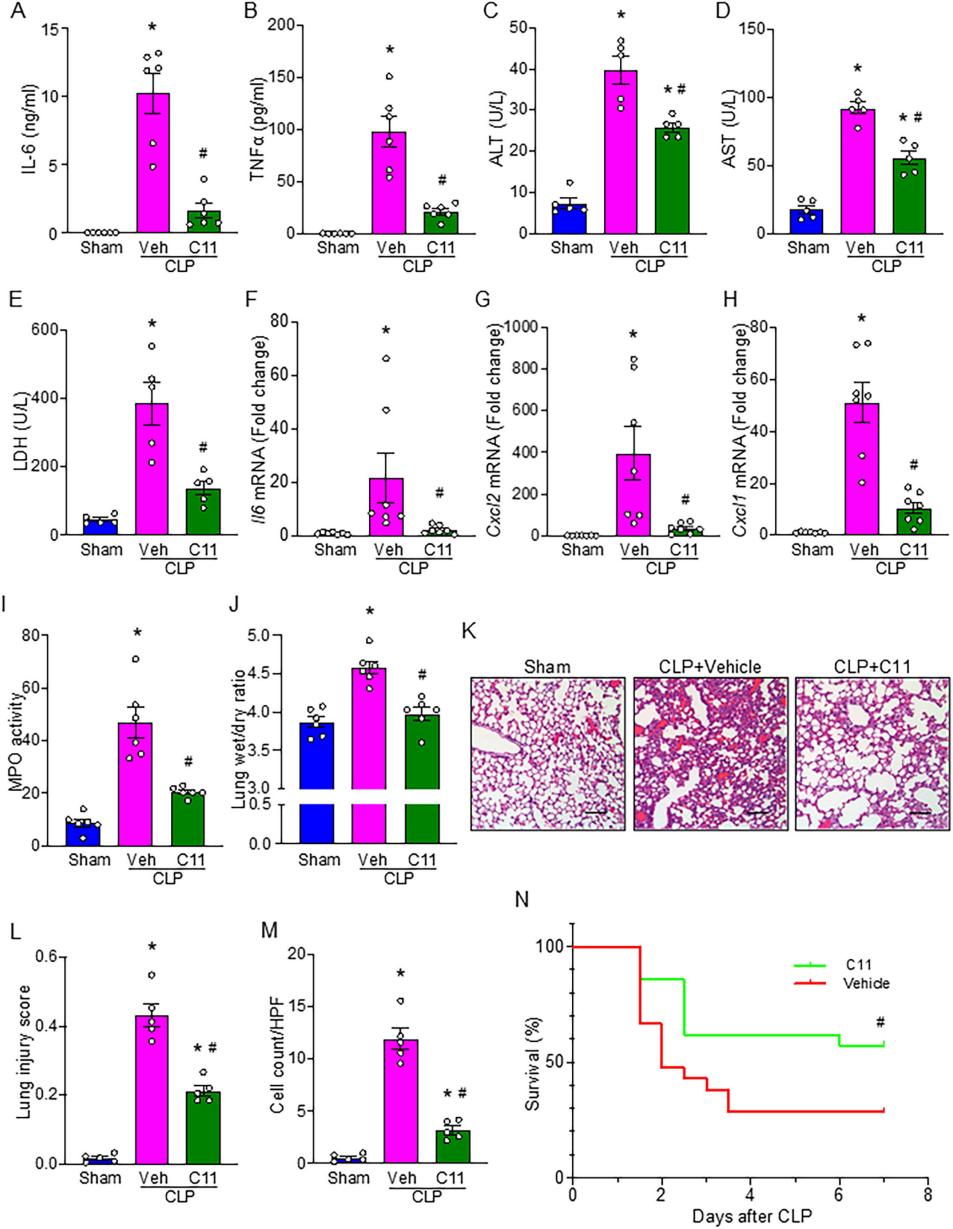
C11 attenuates organ injuries and cytokine production, and improves survival in sepsis. Mice subjected to CLP-induced sepsis were treated with C11 (10 μg/kg) or vehicle (volume equivalent). After 20 h, blood and lung tissue were collected for respective analyses. Plasma was analyzed for systemic **(A)** IL-6 and **(B)** TNFα levels by ELISA. Plasma was analyzed for **(C)** ALT, **(D)** AST, and **(E)** LDH levels by colorimetric assays. Lung tissue mRNA expression of **(F)** IL-6, **(G)** CXCL2, and **(H)** CXCL1 was measured by PCR. **(I)** MPO activity was assessed using colorimetric assays. **(J)** Lung wet to dry weight ratio was determined to evaluate pulmonary edema. **(K)** Representative histological hematoxylin and eosin (H&E) images of lung tissue were shown. Scale bars: 100 μm. **(L)** Lung injury scores were calculated from 0 to 1 based on alveolar and interstitial neutrophil infiltration, hyalinization, protein filling in the airspaces, and wall thickening. **(M)** The number of TUNEL^+^ cells in lung tissues was quantified. Data represent the mean ± SEM (n = 5-7/group). The groups were compared by ANOVA followed by Tukey’s multiple comparisons test to compare multiple groups. *p < 0.05 vs. Sham, ^#^p < 0.05 vs. CLP vehicle. **(N)** Survival rates were analyzed by the Kaplan-Meier estimator using a log-rank test (n = 21/group). ^#^p < 0.05 vs. CLP vehicle. Experiments were performed twice, and all data were analyzed.

To evaluate the therapeutic potential of C11 in regulating neutrophil-mediated B-1a trogocytosis, we used the CLP model to induce sepsis in WT mice and treated them with C11. Systemic levels of TNF-α and IL-6 were significantly elevated in septic mice, whereas C11 treatment significantly reduced these inflammatory cytokines (**Figures 5A, B**). Given the prevalence of secondary organ injury in severe sepsis, we next assessed the impact of C11 on organ injury markers. Blood levels of ALT, AST, and LDH, which were significantly elevated in septic mice, were markedly reduced in the C11-treated group (**Figures 5C–E**). Furthermore, we analyzed lung tissue for signs of inflammation. Lung tissue mRNA levels of IL-6, C-X-C motif chemokine ligand 2 (CXCL2; also known as macrophage inflammatory protein-2, MIP-2), and C-X-C motif chemokine ligand 1 (CXCL1; also known as keratinocyte chemoattractant, KC) were significantly higher in CLP mice compared with sham controls, but these levels were significantly reduced following C11 treatment (**Figures 5F–H**). Likewise, lung MPO activity was significantly elevated in CLP mice but was attenuated by C11 treatment (**Figure 5I**). Additionally, pulmonary edema was evaluated by measuring the lung wet-to-dry ratio, which demonstrated that C11 treatment effectively reduced sepsis-induced pulmonary edema (**Figure 5J**). Histological analyses, including H&E and TUNEL staining, further confirmed severe lung injury in CLP mice. However, C11 treatment significantly mitigated sepsis-induced lung injury, as evidenced by reduced tissue damage and apoptosis (**Figures 5K-M**). Finally, we assessed the survival benefit of C11 treatment in sepsis. C11 administration significantly improved survival from 28.6% to 57.1% in septic mice (**Figure 5N**). Collectively, our findings strongly suggest that C11 effectively preserves B-1a cells, mitigates systemic inflammation and organ injury, including ALI, and confers improved survival in sepsis.

## Discussion

**Figure 6:**
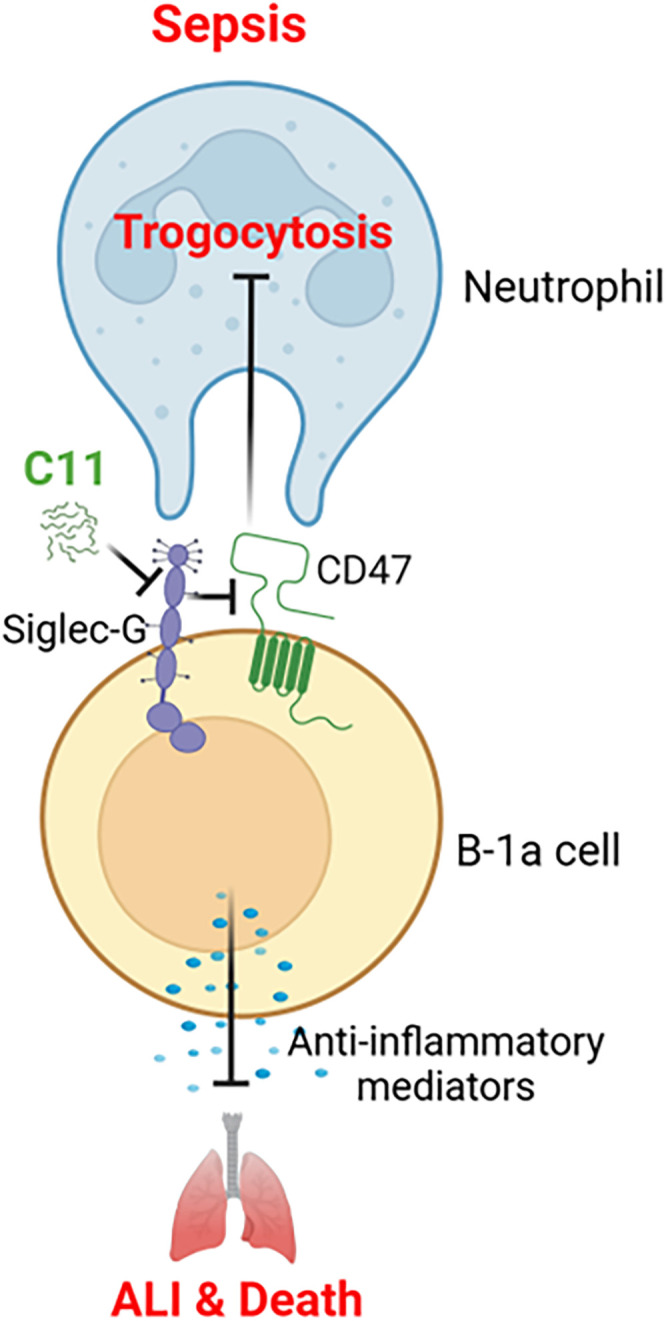
Summary of the findings. B-1a cells are known to protect against sepsis through its regulatory functions, which was significantly reduced in the pleural and peritoneal cavities during sepsis. C11 effectively blocks the Siglec-G and CD47 interaction on B-1a cells in sepsis, thereby inhibiting neutrophil-mediated trogocytosis and preventing the depletion of B-1a cells. This intervention significantly reduced systemic inflammation and improved survival rates in septic mice.

This study reveals a novel pathophysiological mechanism in sepsis and inflammatory diseases, characterized by the loss of B-1a cells, a regulatory B cell subset. We discovered that Siglec-G on B-1a cells may potentially bind to CD47, facilitating neutrophil-mediated trogocytosis and subsequent B-1a cell depletion. Given that B-1a cells play a critical role in controlling hyperinflammation and protection against sepsis (6, 8, 9), their loss may contribute to immune dysregulation and worsening disease outcomes. To address this, we developed C11, a novel peptide specifically designed to disrupt the Siglec-G/CD47 interaction on B-1a cells in sepsis. Treatment with C11 effectively inhibited trogocytosis, preserving B-1a cells, reducing systemic inflammation and ALI, and improving survival in septic mice. These findings suggest that targeting the Siglec-G/CD47 axis on B-1a cells may represent a promising therapeutic approach to mitigate immune dysregulation and improve sepsis (**Figure 6**).

We used two different approaches to rigorously determine the interaction between Siglec-G and CD47 on B-1a cells: *in silico* modeling and BIAcore assays. The validity and translational relevance of this rational design strategy are supported by our previous studies, in which other synthetic peptides targeting molecular interactions attenuated aberrant immune responses and improved outcomes in disease models (11, 31, 32). Siglec-G is characterized by a V-type immunoglobulin-like domain at the N-terminus, which is responsible for recognizing and binding sialic acid-containing ligands, followed by varying numbers of C2-type immunoglobulin-like domains (5, 33). CD47 is a transmembrane glycoprotein belonging to the immunoglobulin superfamily, widely expressed across various cell types and tissues (18, 34). Notably, CD47 is a heavily glycosylated protein with six potential N-linked glycosylation sites at asparagine residues (18). Given that sialylation, the final stage of glycosylation, CD47 possesses a structural basis for binding to Siglec-G through sialylated glycans. These structural properties of CD47 further support the observed Siglec-G/CD47 interaction, suggesting a previously unrecognized regulatory mechanism that contributes to B-1a cell depletion during sepsis. This interaction may impair the SIRPα–CD47 signaling pathway, potentially inhibiting its “don’t-eat-me” function and thereby enhancing neutrophil-mediated trogocytosis.

Trogocytosis has been primarily described in non-phagocytic cells, such as T cells (35, 36). On the other hand, in phagocytes, including neutrophils, trogocytosis has been explored as a novel cancer therapeutic strategy, where neutrophils actively engage in tumor cell killing (20, 37, 38). Studies have indicated that neutrophil-mediated trogocytosis can directly induce cell death through cytoplasmic reduction (20, 38). Despite the increasing interest in neutrophil trogocytosis in oncology, little research has been conducted on its role in sepsis. Previously, we demonstrated that neutrophil-endothelial cell-mediated trogocytosis exacerbates lung injury by altering neutrophil phenotype and function and by promoting the release of pro-inflammatory mediators (39). In this study, we specifically investigated the interaction between neutrophils and B-1a cells within the context of sepsis-induced dysregulation of B-1a cell function and the substantial influx of neutrophils into the pleural and peritoneal cavities. Our data confirm the occurrence of neutrophil-mediated trogocytosis but do not directly establish its impact on B-1a cell viability. However, previous studies have demonstrated that neutrophil trogocytosis induces cell death through cytoplasmic reduction (20, 38). Based on these findings, our data suggest that this mechanism contributes to B-1a cell depletion during sepsis.

We have revealed that peritoneal B-1a cells are significantly reduced in sepsis, consistent with previous studies (11, 40). Additionally, we identified that B-1a cell depletion also occurs in the pleural cavity, suggesting that this phenomenon is not localized but rather represents a systemic process affecting multiple immune compartments. Given the established survival benefits associated with preserving B-1a cells in sepsis (6, 8, 9), understanding the mechanisms driving their reduction is of critical importance for discovering novel therapeutic targets. We discovered C11, a novel peptide, ameliorating B-1a cell depletion in sepsis. We acknowledge the possibility that C11 may have exerted effects beyond inhibiting the Siglec-G/CD47 interaction through other nonspecific mechanisms. However, we rigorously evaluated C11 both physically and functionally using BIAcore analysis and trogocytosis assays, which support its specificity in targeting Siglec-G/CD47 interactions. We also recognize that neutrophil-mediated trogocytosis is not the sole mechanism responsible for B-1a cell depletion in sepsis. Previous studies have proposed multiple contributing factors. For instance, peritoneal macrophages and B-1a cells adhere to the mesothelium in a fibrin-dependent manner, forming multilayered cellular aggregates in an *E. coli* infection model (41). Additionally, peritoneal B-1a cells have been shown to migrate into the spleen following CLP- or LPS-induced sepsis (11, 42). Indeed, our targeted intervention to block the Siglec-G/CD47 interaction and inhibit neutrophil-mediated trogocytosis did not fully restore B-1a cell numbers in sepsis, further supporting the idea that multiple mechanisms contribute to B-1a cell depletion during sepsis. Nevertheless, our translational experiments using a specific peptide targeting the Siglec-G/CD47 interaction demonstrated the physiological significance of this mechanism in sepsis, highlighting its potential impact on disease progression.

In this study, we used only male mice to eliminate the potential influence of sex hormones on disease pathophysiology. We employed a single-dose, simultaneous treatment strategy with C11 at the time of sepsis induction to assess its impact on disease outcomes. Our favorable findings demonstrate that C11 preserves B-1a cells across various compartments, ultimately attenuating sepsis-induced ALI. These results highlight the therapeutic potential of C11 and warrant further investigation into its efficacy in post-sepsis treatment and multi-dose regimens, as well as its pharmacokinetics, including half-life. We previously observed a reduction in B-1a cells in both gut ischemia-reperfusion (I/R) (43) and *E. coli* models (9). Since these preclinical sepsis models also lead to the development of ALI, evaluating the impact of C11 in gut I/R or *E. coli*-induced sepsis could provide further insights into its therapeutic potential. Although we have not yet performed experiments using human cells, sequence analysis reveals that the amino acid sequence at the CD47 interaction site is over 80% conserved between mice and humans and the binding region of murine Siglec-G and its human ortholog Siglec-10 shares more than 70% sequence similarity, suggesting the potential translational applicability of C11 in humans (44).

The effects of C11 may extend beyond preserving B-1a cells to the regulation of neutrophil function through SIRPα-CD47 signaling during sepsis. The SIRPα-CD47 pathway is an attractive potential therapeutic target in sepsis, particularly in the context of neutrophils and other immune cells. Upon engagement with CD47, the tyrosine-phosphorylated sites of SIRPα recruit and activate the src homology-2 (SH2) domain-containing protein tyrosine phosphatase 1 (SHP-1) (45). SHP-1 is predominantly expressed in hematopoietic cells, including neutrophils, and serves as a negative regulator that tempers neutrophil activity, preventing excessive inflammation (46). Indeed, a recent study demonstrated that the loss of neutrophil SHP-1 leads to hyperinflammation and pulmonary hemorrhage in the setting of LPS-induced septic ALI (47). SHP-1 is recruited by SIRPα through binding to immunoreceptor tyrosine-based inhibitory motifs (ITIMs) and functions by dephosphorylating proteins downstream of cytokine receptors, such as TLR4 (46, 48). These findings suggest that SIRPα is a pivotal regulator for preventing hyperactivation of neutrophils, which can exacerbate organ injury during sepsis. The therapeutic effects of C11 in disrupting the Siglec-G/CD47 interaction may not only prevent neutrophil-mediated trogocytosis of B-1a cells, thereby preserving their immunoregulatory function in reducing pro-inflammatory responses and bacterial load but may also enhance SIRPα signaling through CD47 with Siglec-G removed on residual B-1a cells. This suggests that C11 not only protects B-1a cells from depletion but also potentially modulates neutrophil activity, contributing to the mitigation of inflammation and organ injury in sepsis.

In summary, our study revealed a novel mechanism underlying B-1a cell depletion and the crosstalk between neutrophils and B-1a cells during sepsis. The Siglec-G/CD47 interaction on B-1a cells may regulate neutrophil-mediated trogocytosis, contributing to B-1a cell loss. Additionally, the novel peptide C11 presents a potential therapeutic intervention for sepsis by blocking this interaction, thereby preserving B-1a cells and modulating the inflammatory response. Understanding how neutrophil-mediated trogocytosis affects B-1a cells could provide new insights into the mechanisms driving immune dysregulation and organ injury in sepsis.

## Data Availability

The original contributions presented in the study are included in the article/**Supplementary Material**. Further inquiries can be directed to the corresponding authors.
