# Supplementary material for: A novel molecule targeting neutrophil-mediated B-1a cell trogocytosis attenuates sepsis-induced acute lung injury
Source: Front Immunol. 2025 Jun 11;16:1597887. doi: 10.3389/fimmu.2025.1597887 (PMC12187842; doi:10.3389/fimmu.2025.1597887)
Supplement: Supplementary file 1 [file DataSheet1.pdf]

Supplemental Figure 1

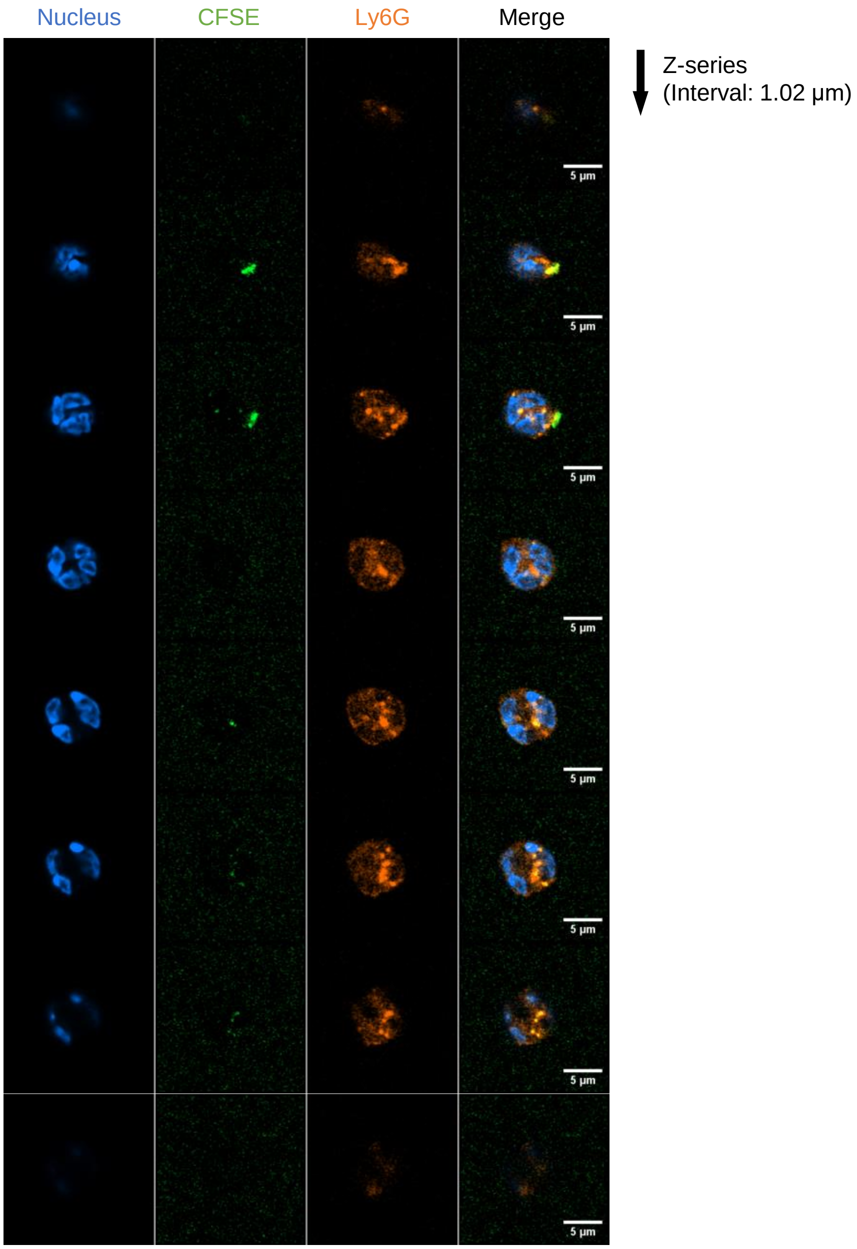

Supplemental Figure 1: Neutrophil-mediated B-1a cell trogocytosis visualized by confocal immunofluorescence microscopy. A montage of Z-stack images was generated using Fiji ImageJ to visualize neutrophil-mediated trogocytosis of B-1a cells. Immunofluorescence staining showed neutrophils stained with Ly6G (orange) and nuclear stain (blue), while fragments of B-1a cells appear CFSE-positive (green). Scale bars: 5 μ m.

Supplemental Figure 2

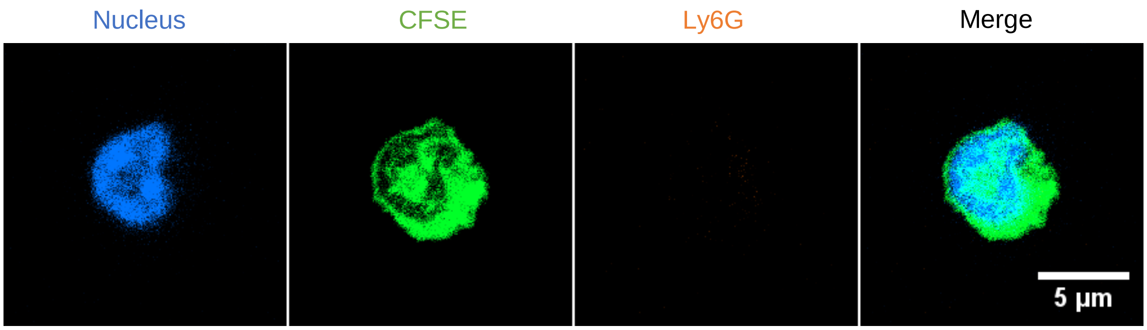

Supplemental Figure 2: Visualization of normal B-1a cells by confocal immunofluorescence microscopy. Immunofluorescence staining shows B-1a cells labeled with CFSE (green) and nuclear staining (blue). These cells were negative for the neutrophil marker Ly6G (orange), confirming that they are B-1a cells and not neutrophils. Scale bars: 5µm.

Supplemental Figure 3

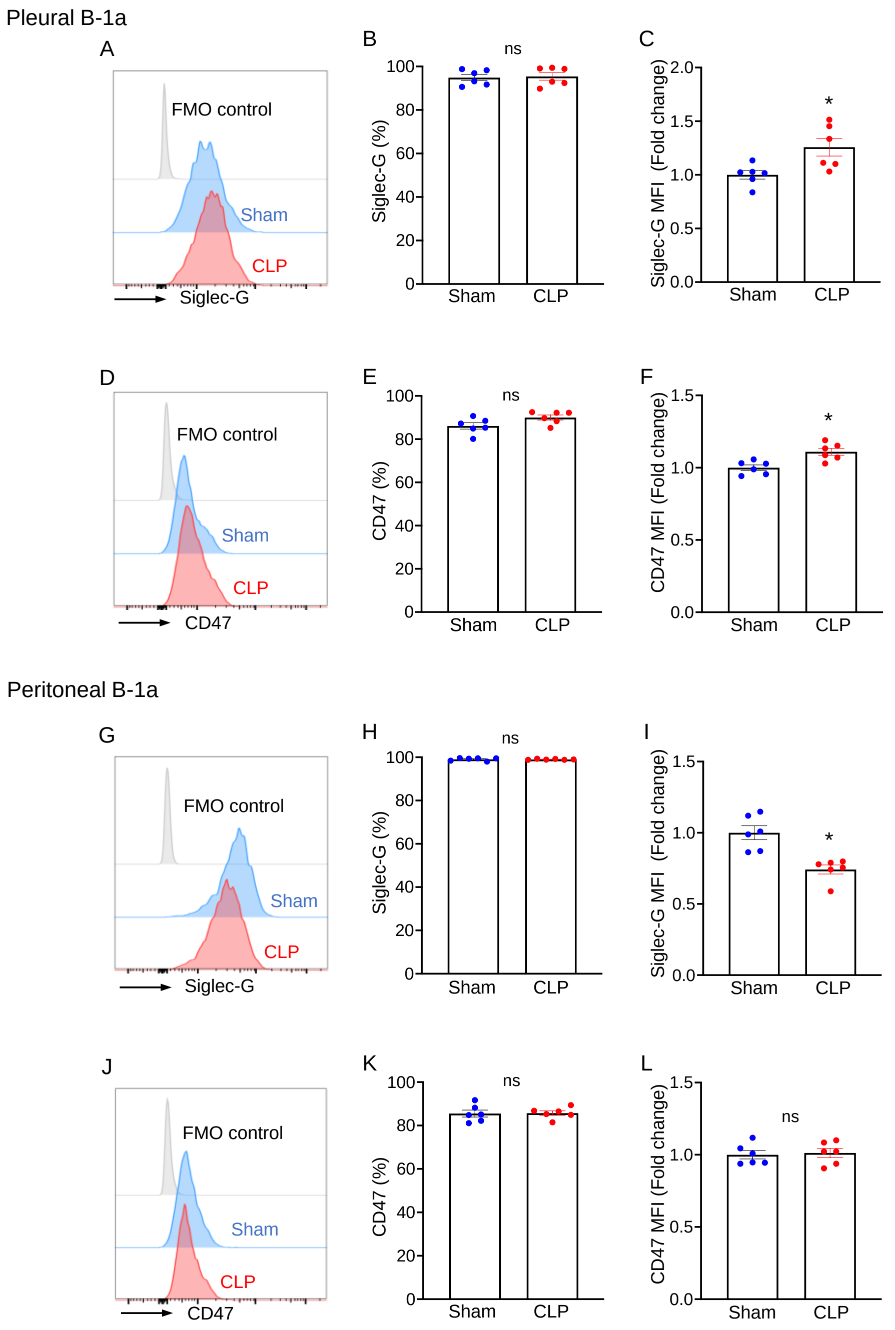

Supplemental Figure 3: B-1a cells expresses Siglec-G and CD47 under both normal conditions and septic conditions. Sepsis was induced in mice using CLP. B-1a cells from **(A-F)** pleural and **(G-L)** peritoneal cavities were collected at 4 h after surgery to determine Siglec-G and CD47 expression levels on B-1a cells. **(B, E, H, K)** The frequency and **(C, F, I, L)** median fluorescence intensity (MFI) of Siglec-G and CD47 expression levels on B-1a cells in the pleural

and peritoneal cavities were analyzed, and **(A, D, G, J)** representative flow cytometry histograms were presented for both sham and CLP. Data represent the mean ± SEM (n = 6/group).

Experiments were performed twice, and all data were analyzed. The groups were compared by Student’s t-test. *$p < 0.05$ vs. Sham.
