# Supplementary material for: A novel molecule targeting neutrophil-mediated B-1a cell trogocytosis attenuates sepsis-induced acute lung injury
Source: Front Immunol. 2025 Jun 11;16:1597887. doi: 10.3389/fimmu.2025.1597887 (PMC12187842; doi:10.3389/fimmu.2025.1597887)
Supplement: Supplementary file 2 [file DataSheet2.pdf]

Supplemental Table 1. Primer pairs for quantitative real-time-PCR

Name	Accession number	Forward Primer (5'- 3')	Reverse Primer (5'- 3')
**IL-6**	NM_031168	CCGGAGAGGAGACTTCACAG	CAGAATTGCCATTGCACAAC
**MIP-2**	NM_009140	CCCTGGTTCAGAAAATCATCCA	GCTCCTCCTTTCCAGGTCAGT
**KC**	NM_008176	GCTGGGATTCACCTCAAGAA	ACAGGTGCCATCAGAGCAGT
**β-actin**	NM_007393	CGTGAAAAGATGACCCAGATCA	TGGTACGACCAGAGGCATACAG

IL-6, Interleukin 6; MIP-2, macrophage inflammatory protein-2; KC, Keratinocyte chemoattractant
